# Effects of mixing alcohol with energy drink on objective and subjective intoxication: results from a Dutch on-premise study

**DOI:** 10.1007/s00213-014-3715-y

**Published:** 2014-08-20

**Authors:** J. C. Verster, J. M. E. Benjaminsen, J. H. M. van Lanen, N. M. D. van Stavel, B. Olivier

**Affiliations:** 1 Utrecht Institute for Pharmaceutical Sciences, Division of Pharmacology, Utrecht University, Universiteitsweg 99, 3584 CG Utrecht, The Netherlands; 2 Centre for Human Psychopharmacology, Swinburne University, Melbourne, VIC 3122 Australia

**Keywords:** Alcohol, Energy drink, Intoxication, Masking

## Abstract

**Background:**

The purpose of this on-premise study was to determine if alcohol mixed with energy drink (AMED) consumption masks the subjective feelings of intoxication when compared to consuming alcohol only.

**Methods:**

The study was conducted on five nights in the city center of Utrecht. *N* = 997 people leaving bars were interviewed about their alcohol consumption with and without energy drinks, for that particular evening and for other occasions. People reporting drug and medication use were excluded (*N* = 84). Subjective intoxication was rated on a 10-point scale. Objective intoxication (breath alcohol concentration, BrAC) was determined with a breath alcohol test. Three groups were identified: (1) the AMED-tonight group (*N* = 185, 20.2 %), (2) the AMED-other-nights group (*N* = 246, 27.1 %), and (3) the no-AMED group (*N* = 482, 52.7 %).

**Results:**

Objective intoxication (BrAC) did not significantly differ (*p* = 0.94) between the AMED-tonight group (0.074 % ± 0.05), AMED-other-nights group (0.073 % ± 0.05), and the no-AMED group (0.074 % ± 0.05). In line, subjective intoxication was not significantly different (*p* = 0.96) between the AMED-tonight group (4.5 ± 2.2), AMED-other-nights group (4.6 ± 2.3), and no-AMED group (4.6 ± 2.2).

Within-subjects comparisons revealed no significant differences in total alcohol consumption between AMED occasions and alcohol only occasions. Regression analyses showed that “gender” (beta = 0.078, *p* = 0.016), “time of testing” (beta = 0.085, *p* = 0.009,) and “BrAC” (beta = 0.574, *p* = 0.0001) together explained 37.7 % of variance of subjective intoxication scores (Cohen’s *f* ^2^ = 0.605). Whether or not subjects consumed energy drinks did not predict subjective intoxication scores.

**Conclusion:**

The data suggests that mixing alcohol with energy drink does not mask subjective intoxication.

## Introduction

Although energy drinks comprise only 1 % of the total non-alcoholic beverage market, these products are becoming increasingly popular (UNESDA 2012). Common energy drinks contain 80 mg caffeine per 250 ml can, comparable to a cup of coffee, but more than for example cola beverages (25 mg per 250 ml) (Verster et al. 2012).

It has been suggested that co-consumption of caffeinated beverages and alcohol may mask the effects of alcohol. The rationale for this assumption is that the stimulant effects of caffeine may counteract the depressant effects of alcohol. If a masking effect truly exists people would feel less intoxicated than they actually are. This would be worrisome as it could have serious consequences. For example, people who are objectively intoxicated may perceive themselves as being less intoxicated or sober and drive a car (Quinn and Fromme 2012). Research to determine if co-consumption of energy drinks and alcohol may result in masking of the intoxicating effect of alcohol is therefore important.

The study that is most commonly cited as supportive of a so-called masking effect caused by consuming alcohol mixed with energy drink (AMED) (Ferreira et al. 2006) reported significant differences on subjective assessments of 4 out of 18 symptoms of alcohol consumption (higher scores of headache, weakness, salivation, and reduced motor coordination). However, these findings were not confirmed in a recent replication study with twice the number of subjects that found no significant difference on any of the 18 symptoms that were examined by Ferreira et al. (Ulbrich et al. 2013). Actual data on the masking effect comes from experimental studies directly comparing subjective intoxication after consuming AMED with alcohol only (Marczinski et al. 2011, 2012, 2013; Peacock et al. 2013). Consistently, none of these four studies found a significant difference on subjective intoxication measures between consuming AMED or alcohol only. In these studies, typical peak breath alcohol concentration (BrAC) ranged from 0.03 to 0.08 % (i.e., the equivalent of 1 to 4 alcoholic drinks), and caffeine levels that varied from 0.6 to 3.57 mg/kg bodyweight (comparable to 0.5 to three 250 ml cans of energy drink). Similar results were found in experimental studies comparing alcohol combined with caffeine versus alcohol only. In these studies, caffeine levels varied from 2 to 5.7 mg/kg bodyweight (comparable to the caffeine amount of 1.75 to 5.7 cans of 250 ml energy drink). Except for one study (Heinz et al. 2013) that did report a small but significant difference (0.5 on a 10 point scale), eight other studies showed that adding caffeine to alcohol does not significantly change subjective intoxication relative to consuming alcohol only (Rush et al. 1993; Azcona et al. 1995; Fillmore and Vogel-Sprott 1999; Fillmore et al. 2002; Marczinski and Fillmore 2003; Marczinski and Fillmore 2006; Howland et al. 2010; Attwood et al. 2012). A recent meta-analysis on these experimental studies confirmed that adding caffeine to alcohol had no significant impact on the judgment of subjective intoxication (Benson et al. 2014). Finally, a longitudinal study following subjects for 14 days (Patrick and Maggs 2013) found that after controlling for estimated blood alcohol concentration (BAC), energy drink use did not predict subjective intoxication. Taken together, these studies suggest that a masking effect does not exist.

However, it is important to take into account that higher BrAC levels have been reported in real-life settings than those permitted in controlled experimental studies. This is evident from surveys examining AMED and alcohol consumption (e.g., De Haan et al. 2012a, b), but also from observational studies (e.g., Hesse and Tutenges 2009). On-premise studies can provide additional information of alcohol consumers with higher BrACs. In on-premise studies, participants are interviewed during or immediately after their drinking session, for example when leaving the pub. On-premise studies allow comparing people who consumed energy drinks on that particular evening with those who did not, and determine whether they differ in perceived subjective intoxication. Hence, these studies are ideal to directly compare measurements of objective and subjective intoxication. Up to now, three on-premise studies examined alcohol and energy drink consumption of bar patrons (Thombs et al. 2010a, b; Wells et al. 2013), but unfortunately, subjective intoxication was not assessed in these studies.

Therefore, the aim of the current study was to examine both objective and subjective intoxication in an on-premise setting, and to determine if mixing alcohol with energy drink masks the subjective feeling of intoxication when compared to consuming alcohol only. A disadvantage of previous on-premise studies is that they presented only between-group comparisons. That is, alcohol consumption of AMED consumers was compared to other people who consume alcohol only. Studies applying between-group comparisons often report significantly increased total alcohol consumption in the AMED group when compared to the alcohol only group. This difference, however, is most likely not caused by co-consumption of energy drinks, but observed because AMED consumers differ in many ways from people who consume alcohol only, including demographics such as gender and age, personality, level of risk-taking behavior, and their alcohol consumption patterns (e.g., O’Brien et al. 2008; Woolsey et al. 2010; Berger et al. 2011; Brache and Stockwell 2011; De Haan et al. 2012b; Cheng et al. 2012; Miller 2012; Verster et al. 2012; Snipes and Benotsch 2013; Wells et al. 2013). Therefore, the current on-premise study also included within-subjects comparisons to enable comparing alcohol consumption on occasions on which they consume AMED with occasions on which they consume alcohol only. Given the scientific evidence from experimental trials, it is hypothesized that mixing alcohol with energy drink does not affect objective and subjective intoxication.

## Methods

The aim of the on-premise study was to recruit *N* = 1,000 participants, aged 18–30 years old, who consumed alcohol on that evening. Based on recent survey results in The Netherlands (De Haan et al. 2012b), this sample size is sufficient to include an adequate number of AMED consumers. The study was conducted in October 2012 in Utrecht, The Netherlands. The study protocol was reviewed and approved by the Medical Ethics Committee Twente. Venue owners and local police were informed about the study in advance.

### Procedure

Study nights were Wednesday to Saturday. Since participants could be intoxicated and/or on their way home, the length of the interview was limited to 5 min. Twelve trained investigators were divided in teams of two testers. They were located at the entrance of bars and clubs in the city center of Utrecht to recruit potential participants. They approached people who were leaving the venue, between 11:00 p.m. and 05:00 a.m. As soon as the potential participants gave verbal consent to take part in the study, they were interviewed and breath alcohol levels were tested. One experimenter determined the breath alcohol concentration (BrAC) using an Alcohit breath analyzer, while the other experimenter conducted the interview. Participants were not informed about their BrAC, in order not to influence them when estimating their level of intoxication. Subjective intoxication was estimated on a scale ranging from 0 (sober) to 10 (completely drunk).

**Fig. 1:**
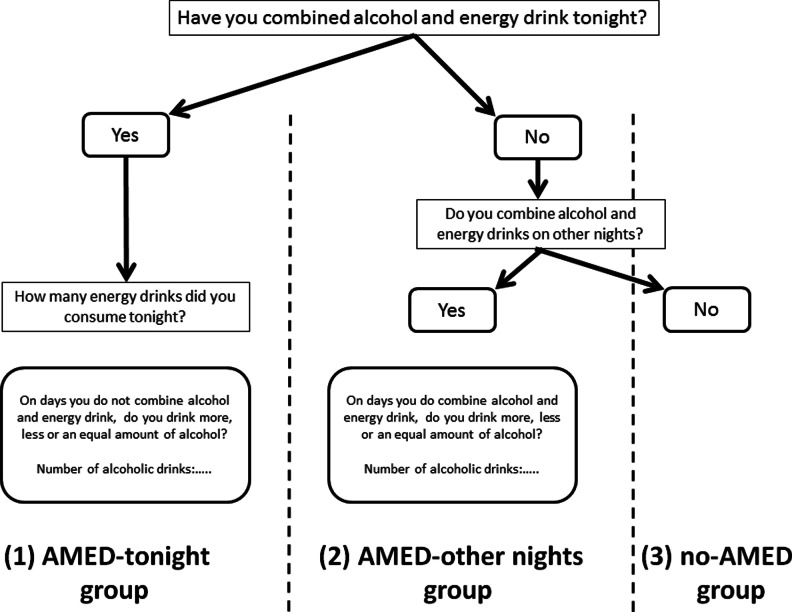
Flow chart of consumption questions. *AMED* alcohol mixed with energy drink

Demographics were limited to sex and age of the participant. Participants reported the number of alcoholic drinks they consumed that evening, and the start and stop time of drinking. Smoking, medicinal drugs, and illicit drug use were assessed, and all participants answered the 3-item AUDIT-C (Aertgeerts et al. 2001), to identify potential risk for alcohol use disorder. Furthermore, participants were asked whether they had consumed both alcohol and energy drinks that evening, or do so on other occasions than the night of the study. Energy drinks were defined as 250 ml cans of regular brands such as Red Bull (containing 80 mg of caffeine). The flowchart of consumption questions is shown in Fig. 1.

Based on their consumption pattern, subjects were allocated to one of the following three groups: (1) AMED-tonight, (2) AMED-other nights, or (3) no-AMED (alcohol only). Within-subjects comparisons regarding alcohol consumption were made in the AMED-tonight and AMED-other nights group by comparing with alcohol only or AMED consumption, respectively, on the other night. First, we asked whether people consume “more”, “less”, or “equal” amounts of alcohol on the other occasion. Second, we asked the participant to estimate the actual number of alcoholic drinks for the other occasion. These questions enabled to determine if within-subjects total alcohol consumption differs between AMED and alcohol only occasions.

Finally, it was asked if participants planned to continue drinking after the interview or planned to go home. If they answered traveling home by car and intended to drive themselves, they were advised not to do so, and it was offered to call a taxi. As soon as the interview was finished, the interviewers approached the next potential participant that was leaving the bar.

### Statistical analyses

Statistical analyses were performed using SPSS, version 20. Means, standard deviation, and frequency distributions were computed for all variables. Three groups of alcohol consumers were identified: (1) the AMED-tonight group (people who mixed alcohol and energy drink on that occasion), (2) the AMED-other-nights group (people who do consume AMED, but not on the evening of the interview), and (3) the no-AMED group (people who never mix alcohol with energy drink). For the between-group analyses, data was analyzed using analysis of variance (ANOVA). Posthoc Tukey-HSD corrected for multiple comparisons. Chi-square tests were used to analyze categorical data. Within-subject comparisons compared total alcohol consumption on occasions when individuals do and do not combine alcohol with energy drink. The analyses were performed using dependent *t* tests. To determine which variables predict subjective intoxication, linear regression analyses were conducted. Results were considered significant if *p* < 0.05. Effect sizes were reported for significant mean differences.

## Results

*N* = 997 subjects participated in the study. Although not formally recorded, response rate was estimated at 80 %. People reporting drug and medication use were excluded (*N* = 84); *N* = 913 were included. Three groups were identified: (1) the AMED-tonight group (*N* = 185, 20.2 %), (2) the AMED-other-nights group (*N* = 246, 27.1 %), and (3) the no-AMED group (*N* = 482, 52.7 %). The male/female ratio was 44.9 %/55.1 % (*p* = 0.003). On average, subjects from the no-AMED group were slightly but significantly older (21.8 ± 3.1, *p* = 0.0001) when compared to the AMED-tonight group (20.7 ± 2.7, *p* = 0.0001, Cohen’s *d* = 0.367) and AMED-other night groups (20.7 ± 2.8, *p* = 0.0001, Cohen’s *d* = 0.336). The AMED-other night group scored significantly higher on the AUDIT-C (8.2 ± 2.0) when compared to the AMED-tonight group (7.8 ± 2.1, *p* = 0.001, Cohen’s *d* = 0.194) and no-AMED group (7.6 ± 2.1, *p* = 0.001, Cohen’s *d* = 0.289).

### Between-group comparisons

**Fig. 2:**
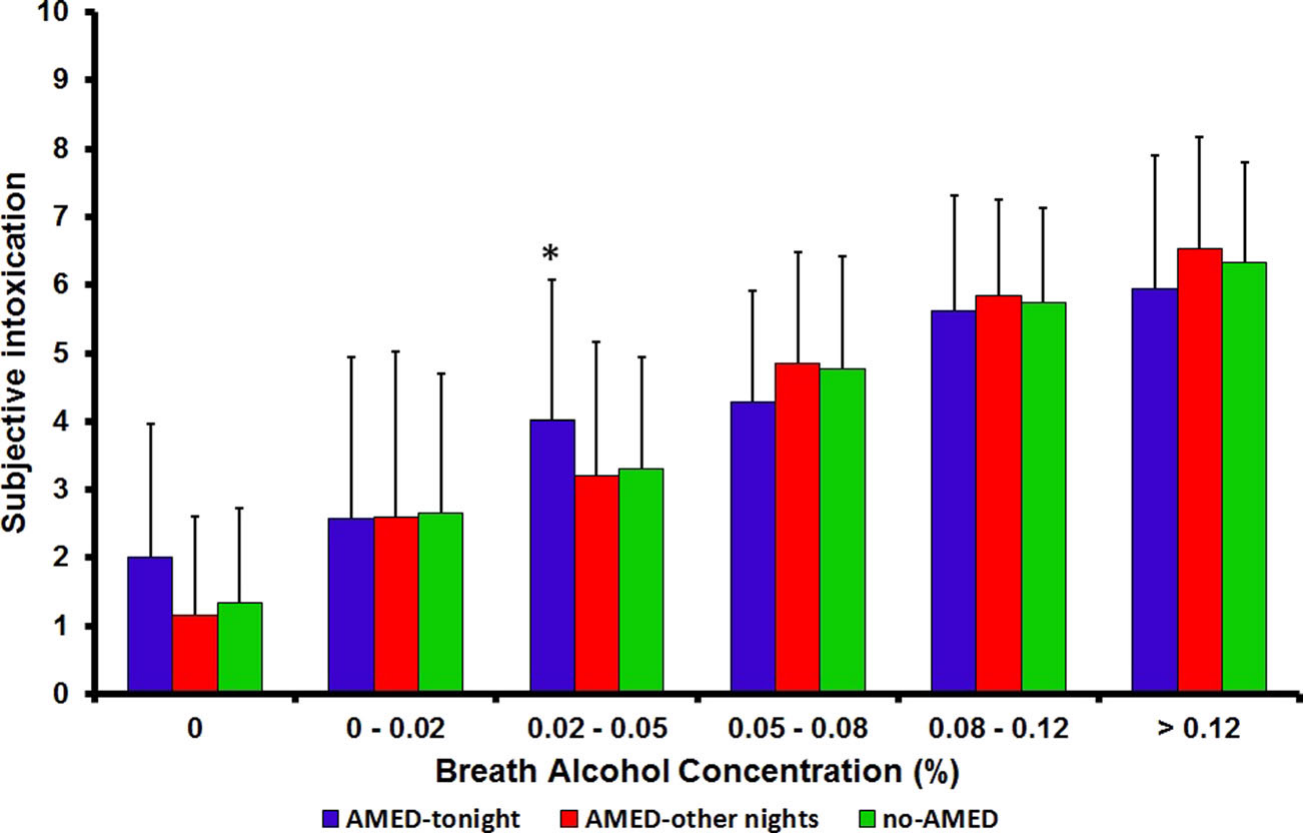
(Mean ± SD) subjective intoxication scores for different BrAC ranges. *p < 0.05, significant differences. *BrAC* breath alcohol concentration, *AMED* alcohol mixed with energy drink, *SD* standard deviation

Objective intoxication (BrAC) did not significantly differ (*p* = 0.940) between the AMED-tonight group (0.074 % ± 0.05), AMED-other-nights group (0.073 % ± 0.05), and the no-AMED group (0.074 % ± 0.05). In line, subjective intoxication was not significantly different (*p* = 0.960) between the AMED- tonight group (4.5 ± 2.2), AMED-other-nights group (4.6 ± 2.3), and no-AMED group (4.6 ± 2.2). Non-significant differences were found consistently for different BrAC ranges, except for significantly a higher subjective intoxication score for the AMED-tonight group in the BAC 0.02–0.05 % range than the AMED-other night group (*p* = 0.032, Cohen’s *d* = 0.556) and no-AMED group (*p* = 0.002, Cohen’s *d* = 0.435) (see Fig. 2).

Between-group comparisons revealed that total drinking time did not significantly differ between the groups (*p* = 0.790). Overall, the AMED-tonight group consumed significantly more alcoholic drinks than the no-AMED group (+1.3 drinks, *p* = 0.020, Cohen’s *d* = 0.223), caused by consuming significantly more alcohol before going out (+1.1 drinks, Cohen’s *d* = 0.270). The AMED-tonight group smoked significantly more cigarettes than the no-AMED group (+2.3 cigarettes, *p* = 0.012, Cohen’s *d* = 0.399). No significant differences were found between the AMED-tonight and AMED-other-nights group. The AMED-tonight group consumed on average 2.0 ± 1.8 energy drinks. A summary of the data is presented in Table 1.

**Table 1:** Between-group comparisons

	AMED-tonight	AMED-other nights	Alcohol only
	(*N* = 185)	(*N* = 246)	(*N* = 482)
BrAC (%)	0.074 (0.05)	0.073 (0.05)	0.074 (0.05)
Subjective intoxication score	4.5 (2.2)	4.6 (2.3)	4.6 (2.2)
Alcoholic drinks before going out	4.9 (5.1)*	4.8 (4.6)	3.8 (3.6)
Alcoholic drinks on-premise	5.2 (5.1)	4.6 (4.4)	5.0 (4.5)
Total alcoholic drinks	10.1 (6.8)*	9.4 (6.3)	8.8 (5.4)
Start time alcohol consumption	9:03 pm (2:05)	9:25 pm (2:09)	9:10 pm (2:08)
Stop time alcohol consumption	2:25 am (1:44)	2:14 am (1:18)	2:16 am (1:29)
Total drinking time (hours, minutes)	5:09 (2:34)	4:56 (2:34)	5:05 (2:38)
Energy drinks before going out	1.1 (1.2)	0	0
Energy drinks on-premise	0.9 (1.6)	0	0
Total energy drinks	2.0 (1.8)	0	0
Number (%) smokers	81 (43.8 %)	116 (47.2 %)*	185 (38.4 %)
Total cigarettes smoked	9.7 (6.8)*	8.4 (6.4)	7.4 (5.3)

Mean and standard deviation (*between brackets*) are shown. Please note that in The Netherlands alcoholic drinks all have the same standardized amount of 10 g alcohol. An energy drink unit was defined as a 250 ml can

*BrAC* breath alcohol concentration

*p < 0.05, significant differences with the Alcohol Only Group

### Within-subjects comparisons

Within-subject comparisons revealed that the majority of people from the AMED-tonight group and AMED-other-nights consume the same number of alcoholic drinks on alcohol only and AMED occasions. The AMED-tonight group consumed 10.1 (±6.8) alcoholic drinks on the night when they were interviewed versus 9.4 (±5.9) on alcohol only occasions (*p* = 0.148, Cohen’s *d* = 0.055). The AMED-other-nights group consumed 9.4 (±6.3) when they were interviewed (alcohol only) versus 9.8 (±6.0) drinks on AMED occasions (*p* = 0.360, Cohen’s *d* = 0.106).

### Regression analysis

A regression analysis was conducted to determine if group membership and other variables (gender, age, time of testing, and BrAC) could predict subjective intoxication. The analyses revealed that belonging to the AMED-tonight, AMED-other night, or no-AMED group did not significantly predicted subjective intoxication. Conducting a subsequent analysis without “group” as predictor variable revealed that also “age” could be deleted from the model. The remaining predictors were “gender” (beta = 0.078, *p* = 0.016), “time of testing” (beta = 0.085, *p* = 0.009), and “BrAC” (beta = 0.574, *p* = 0.0001), which together explained 37.7 % of variance of subjective intoxication scores (*F* = 126.02, df = 3/617, *p* < 0.0001, Cohen’s *f* ^2^ = 0.605). The regression analysis was also conducted with BrAC replaced by the total number of alcoholic drinks consumed. A significant model was found, in which the variables “gender” (beta = −0.104, *p* = 0.297), “age” (beta = 1.209, *p* = 0.227), “time of testing” (beta = 0.101, *p* = 0.001), and “total alcohol consumed” (beta = 0.563, *p* = 0.0001) explained 35.0 % of variance of the subjective intoxication scores (*F* = 115.50, df = 4, 848, Cohen’s *f* ^2^ = 0.538). Again, the factor “group” (i.e., whether or not participants consumed AMED) did not significantly contribute to the model.

**Fig. 3:**
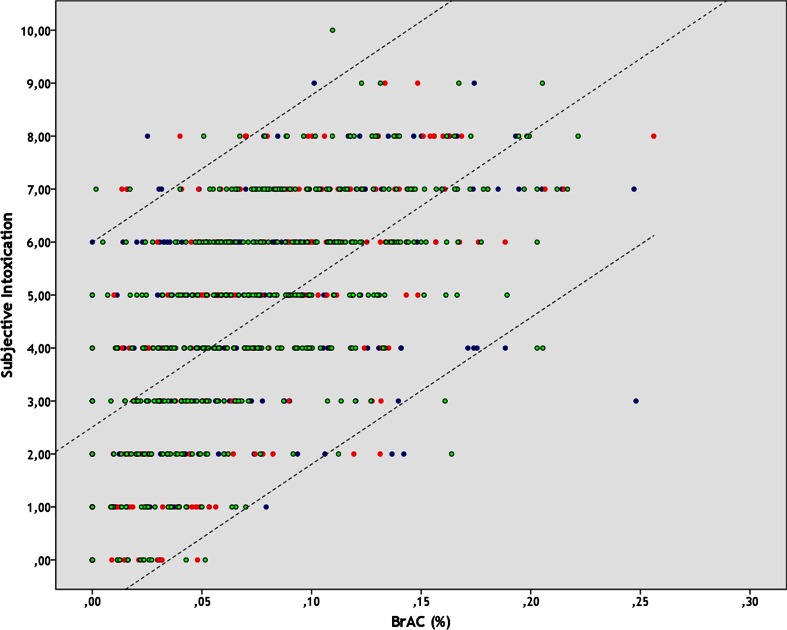
The relationship between objective intoxication (BrAC) and subjective intoxication. Aggregated data is shown for subjects from the AMED-tonight group (*blue*), AMED-other nights group (*red*), and Alcohol Only group (*green*). *Dashed lines* represent the significant correlation between subjective and objective intoxication (*R* ^2^ = 38.1 %) and the corresponding 95 % confidence interval. *BrAC* breath alcohol concentration

Figure 3 illustrates that BrAC significantly (*p* = 0.0001) correlated with scores of subjective intoxication (*r* = 0.617), but this association was stronger in men (*r* = 0.626) when compared to women (*r* = 0.574). Time of testing also significantly correlated with scores of subjective intoxication (*r* = 0.267, *p* = 0.0001).

### Reported activities after the interview

At the time of testing, 51.8 % of the AMED-other night group and 48.0 % of the no-AMED group reported continuing drinking alcohol after completion of the interview. In contrast, a significant smaller percentage of the AMED-tonight group reported continuing drinking alcohol (36.7 %, *p* < 0.05). Of those who reported going home (85 % of the total sample), *N* = 34 intended to drive a car. They were equally divided among participants from the AMED-tonight group (*N* = 9, 4.9 %), AMED-other night group (*N* = 11, 4.5 %), and no-AMED group (*N* = 14, 3.0 %). No significant differences were found between the three groups (*p* = 0.388). The majority of the subjects who intended driving home by car (70.6 %) had a BrAC below 0.05 %, i.e., the legal limit for driving in The Netherlands.

## Discussion

In real-life circumstances, this on-premise study confirmed findings from the vast majority of experimental studies that a so-called masking effect does not exist when consuming AMED (Benson et al. 2014). Thus, our data suggests that mixing alcohol with energy drink does not create the subjective perception of being less intoxicated compared to consuming alcohol only.

Regression analyses revealed that the level of subjective intoxication is significantly predicted by BrAC, time of testing during the night out, and gender. These factors together determine 37.7 % of the subjective intoxication score. Not surprisingly, BrAC was by far the strongest predictor of subjective intoxication; those with a higher BrAC usually reported higher scores of subjective intoxication (see Fig. 3). Female participants, and those tested later at night (which often also had higher BrACs), were worse in estimating their level of intoxication compared to men and those tested earlier at night, respectively. However, whether or not participants combined alcohol with energy drink on that night out did not significantly predict subjective intoxication scores.

Between-group comparisons revealed that there are no overall significant differences in both objective (BrAC) and subjective intoxication between occasions when they consume AMED and those when they consume alcohol only. When examining subjective intoxication for different BAC ranges, no significant differences were found between the groups, except for a significant difference for a low BAC range (0.02–0.05 %) at which AMED consumers had significantly *higher* scores than the other groups (i.e., the opposite of what was expected if a masking effect would exist). However, it should be taken into account that drinkers are generally not considered to be intoxicated at these BAC levels. In general, cutoff values for alcohol intoxication correspond to the usual legal limits to drive a car which is BAC 0.05 or 0.08 %. Hence, the relevance of this finding is unclear. The between-group comparisons confirmed however that AMED consumers differ from alcohol only consumers in several aspects (Verster et al. 2012). For example, small but significant differences in total alcohol consumption and number of cigarettes smoked were found. Unfortunately, we did not assess pre-on-premise drinking time in the current study. A difference in pre-drinking time between the groups may have explained why similar BrAC readings were observed while the AMED group did consumed more alcohol than the other groups.

The observed differences are in line with previous research applying between-group comparisons (e.g., Woolsey et al. 2010; Brache and Stockwell 2011; De Haan et al. 2012a, b). One can speculate about the causes of the observed differences between AMED consumers and those who consume alcohol only, but they may be related to differences in demographics, personality, and risk-taking profiles of AMED consumers (e.g., O’Brien et al. 2008; Woolsey et al. 2010; Berger et al. 2011; Brache and Stockwell 2011; De Haan et al. 2012b; Cheng et al. 2012; Miller 2012; Verster et al. 2012; Snipes and Benotsch 2013; Wells et al. 2013). Future research could further investigate these differences.

However, to answer the question whether or not alcohol consumption is increased when consuming AMED versus alcohol only, within-subjects comparisons among AMED consumers should be made. The within-subjects comparisons in this study revealed no significant difference in total alcohol consumption between AMED occasions and occasions on which alcohol only is consumed. This finding is in line with results from surveys that also applied within-subjects comparisons. Whereas some studies found small but significant increases in alcohol consumption when consuming AMED (Price et al. 2010; Brache and Stockwell 2011; Peacock et al. 2012), other studies found small but significant decreases in overall alcohol consumption when consuming AMED (Woolsey et al. 2010; De Haan et al. 2012b). A recent meta-analysis on these five studies (i.e., within-subjects comparison among *N* = 1,814 AMED consumers) revealed that that co-consumption of energy drink has no significant impact on total alcohol consumption (*p* = 0.669, 95%CI −0.183 to 0.285) (Verster et al. 2013).

The major strength of the current study is that the data was collected in an ecologically valid setting, i.e., a bar district. As participants’ drinking behavior was not influenced by upfront study restrictions or instructions, real-life data was obtained, including higher BrAC levels than are usually targeted in experimental studies because of ethical reasons. In addition, the study had a sufficient sample size to draw reliable conclusions concerning potential predictors of subjective intoxication.

The fact that most participants were young and students may be seen as a limitation for the generalizability of our study. On the other hand, consumers between 18 and 30 years old represent a large segment of energy drink consumers. Also, the fact that the study was performed in the Utrecht bar district may limit the generalizability to other countries. However, it is not likely that cultural differences may affect ratings of subjective intoxication in countries where alcohol consumption is a common part of social life. Nevertheless, it would be interesting to replicate this study in other countries to examine potential cross-cultural differences.

Although not all participants were severely intoxicated, the level of intoxication may have had an impact on the answers provided by the participants. To limit the effort for participants, the interview was of short duration and consisted of simple questions. For example, subjective intoxication could be rated from 0 (absent) to 10 (extreme). BrAC was objectively measured with a breath alcohol analyzer. It is unlikely that recall bias had a significant effect on the assessments of total alcohol consumption as we used a within-subject design to compare the different occasions. First, there is no reason to assume that participants recall alcohol consumption for occasion A differently than for occasion B. Second, the results confirmed that there were no differences in total alcohol consumption. It should be noted that questions of the within-subjects comparison comprised the on-premise evening versus another occasion in the past. It can be hypothesized that recall bias may have had an influence on this within-subjects comparison. However, this was not evident from our data: the number of drinks reported by the AMED-tonight and AMED-other night groups for both occasions did not differ significantly from each other.

Finally, it may also be interesting to compare energy drink with other non-alcoholic beverages that are used as mixer. Within-subjects comparison may reveal if mixing alcohol with energy drink has a different effect on overall alcohol consumption when compared to other mixers such as cola or tonic. It is evident from the current study that the majority of people consume alcohol at levels that are higher than recommended (e.g., many have a BrAC that does not allow them to drive a car), irrespective of whether they consume AMED. Future research should therefore aim at the reduction of alcohol consumption per se.

In conclusion, the current study adds to the growing evidence that mixing alcohol with energy drink does not mask subjective intoxication.

## References

[CR1] Aertgeerts B, Buntinx F, Ansoms S (2001). Screening properties of questionnaires and laboratory tests for the detection of alcohol abuse or dependence in a general practice population. Br J Gen Pract.

[CR2] Attwood AS, Rogers PJ, Ataya AF, Adams S, Munafò MR (2012). Effects of caffeine on alcohol-related changes in behavioural control and perceived intoxication in light caffeine consumers. Psychopharmacol.

[CR3] Azcona O, Barbanoj MJ, Torrent J, Jané F (1995). Evaluation of the central effects of alcohol and caffeine interaction. Br J Clin Pharmacol.

[CR4] Benson S, Verster JC, Alford C, Scholey A (2014) Effects of mixing alcohol with caffeinated beverages on subjective intoxication: a systematic review and meta-analysis. Neuroscience & Biobehavioral Reviews 10.1016/j.neubiorev.2014.07.008 25036891

[CR5] Berger LK, Fendrich M, Chen H-Y, Arria AM, Cisler RA (2011). Sociodemographic correlates of energy drink consumption with and without alcohol: results of a community survey. Addict Behav.

[CR6] Brache K, Stockwell T (2011). Drinking patterns and risk behaviors associated with combined alcohol and energy drink consumption in college drinkers. Addict Behav.

[CR7] Cheng W-J, Cheng Y, Huang M-C, Chen C-J (2012). Alcohol dependence, consumption of alcoholic energy drinks and associated work characteristics in the Taiwan working population. Alcohol Alcohol.

[CR8] De Haan L, de Haan H, Olivier B, Verster JC (2012). Alcohol mixed with energy drinks: methodology and design of the Utrecht student survey. Int J Gen Med.

[CR9] De Haan L, de Haan HA, van der Palen J, Olivier B, Verster JC (2012). The effects of consuming alcohol mixed with energy drinks (AMED) versus consuming alcohol only on overall alcohol consumption and alcohol-related negative consequences. Int J Gen Med.

[CR10] Ferreira SE, de Mello MT, Pompéia S, de Souza-Formigoni MLO (2006). Effects of energy drink ingestion on alcohol intoxication. Alcohol Clin Exp Res.

[CR11] Fillmore MT, Vogel-Sprott M (1999). An alcohol model of impaired inhibitory control and its treatment in humans. Exp Clin Psychopharmacol.

[CR12] Fillmore MT, Roach EL, Rice JT (2002). Does caffeine counteract alcohol-induced impairment? The ironic effects of expectancy. J Stud Alcohol.

[CR13] Heinz AJ, de Wit H, Lilje TC, Kassel JD (2013). The combined effects of alcohol, caffeine, and expectancies on subjective experience, impulsivity, and risk-taking. Exp Clin Psychopharmacol.

[CR14] Hesse M, Tutenges S (2009). Evening experiences versus drinking indicators as predictors of hangover on a summer holiday. Am J Addict.

[CR15] Howland J, Rohsenow DJ, Arnedt JT (2010). The acute effects of caffeinated versus non-caffeinated alcoholic beverage on driving performance and attention/reaction time. Addiction.

[CR16] Marczinski CA, Fillmore MT (2003). Dissociative antagonistic effects of caffeine on alcohol-induced impairment of behavioral control. Exp Clin Psychopharmacol.

[CR17] Marczinski CA, Fillmore MT (2006) Clubgoers and their trendy cocktails: implications of mixing caffeine into alcohol on information processing and subjective reports of intoxication. Exp Clin Psychopharmacol 14:450–458 10.1037/1064-1297.14.4.450 17115872

[CR18] Marczinski CA, Fillmore MT, Bardgett ME, Howard MA (2011). Effects of energy drinks mixed with alcohol on behavioral control: risks for college students consuming trendy cocktails. Alcohol Clin Exp Res.

[CR19] Marczinski CA, Fillmore MT, Henges AL, Ramsey MA, Young CR (2012). Effects of energy drinks mixed with alcohol on information processing, motor coordination and subjective reports of intoxication. Exp Clin Psychopharmacol.

[CR20] Marczinski CA, Fillmore MT, Henges AL, Ramsey MA, Young CR (2013). Mixing an energy drink with an alcoholic beverage increases motivation for more alcohol in college students. Alcohol Clin Exp Res.

[CR21] Miller KE (2012). Alcohol mixed with energy drink use and sexual risk-taking: casual, intoxicated, and unprotected sex. J Caffeine Res.

[CR22] O’Brien MC, McCoy TP, Rhodes SD, Wagoner A, Wolfson M (2008). Caffeinated cocktails: energy drink consumption, high-risk drinking, and alcohol-related consequences among college students. Acad Emerg Med.

[CR23] Patrick ME, Maggs JL (2013). Energy drinks and alcohol: links to alcohol behaviors and consequences across 56 days. J Adolesc Health.

[CR24] Peacock A, Bruno R, Martin FH (2012). The subjective physiological, psychological, and behavioral risk-taking consequences of alcohol and energy drink co-ingestion. Alcohol Clin Exp Res.

[CR25] Peacock A, Bruno R, Martin FH, Carr A (2013). The impact of alcohol and energy drink consumption on intoxication and risk-taking behavior. Alcohol Clin Exp Res.

[CR26] Price SR, Hilchey CA, Darredeau C, Fulton HG, Barrett SP (2010) Energy drink co-administration is associated with increased reported alcohol ingestion. Drug Alcohol Rev 29:331–333 10.1111/j.1465-3362.2009.00163.x 20565526

[CR27] Quinn PD, Fromme K (2012). Event-level associations between objective and subjective alcohol intoxication and driving after drinking across the college years. Psychol Addict Behav.

[CR28] Rush CR, Higgins ST, Hughes JR, Bickel WK, Wiegner MS (1993). Acute behavioral and cardiac effects of alcohol and caffeine, alone and in combination, in humans. Behav Pharmacol.

[CR29] Snipes DJ, Benotsch EG (2013). High-risk cocktails and high-risk sex: examining the relation between alcohol mixed with energy drink consumption, sexual behavior, and drug use in college students. Addict Behav.

[CR30] Thombs D, Rossheim M, Barnett T, Weiler R, Moorhouse M, Coleman B (2010). Is there a misplaced focus on AmED? Associations between caffeine mixers and bar patron intoxication. Drug Alcohol Depend.

[CR31] Thombs D, O’Mara R, Tsukamotot M, Rossheim M, Weiler R, Merves M, Goldberger B (2010). Event level analysis of energy drink consumption and alcohol intoxication in bar patrons. Addict Behav.

[CR32] Ulbrich A, Hemberger SH, Loidl A, Dufek S, Pablik E, Fodor S, Herle M, Aufricht C (2013). Effects of alcohol mixed with energy drink and alcohol alone on subjective intoxication. Amino Acids.

[CR33] UNESDA (2012) UNESDA Code for the labelling and marketing of energy drinks. Available at: www.unesda.org

[CR34] Verster JC, Aufricht C, Alford C (2012). Energy drinks mixed with alcohol: misconceptions, myths, and facts. Int J Gen Med.

[CR35] Verster JC, Benson S, Scholey A, Alford C (2013). Meta-analysis comparing alcohol consumption on alcohol mixed with energy drink (AMED) occasions versus alcohol only occasions. Drug Alcohol Rev.

[CR36] Wells BE, Kelly BC, Pawson M, LeClair A, Parsons JT, Golub SA (2013). Correlates of concurrent energy drink and alcohol use among socially active adults. Am J Drug Alcohol Abuse.

[CR37] Woolsey C, Waigandt A, Beck NC (2010). Athletes and energy drinks: reported risk-taking and consequences from the combined use of alcohol and energy drinks. J Appl Sport Psychol.

